# Supplementary material for: The impact of COVID-19 on health financing in Kenya
Source: PLOS Glob Public Health. 2023 Oct 27;3(10):e0001852. doi: 10.1371/journal.pgph.0001852 (PMC10610457; doi:10.1371/journal.pgph.0001852)
Supplement: S1 Appendix — (PDF) [file pgph.0001852.s001.pdf]

1. **Data collection tools**

Tool 1: Interview guide

Key informant profile:

1. Could you introduce yourself and your organization?

A. COVID-19 and Purchasing arrangements

General

1. Could you describe how healthcare services consumed by residents in your country are paid for in your country and who pays for them?
2. Could you describe how COVID-19 healthcare services provided to residents are paid for in your country? (e.g. COVID-19 testing, isolation and treatment)?
   a. Does the government pay for all or part of these services on behalf of patients?

What to purchase

3. Is there a defined set/list of healthcare services that the government provides or pays for its residents?
4. If yes to 3 above, did this set of services adequately accommodate or include all or some of the COVID-19 healthcare services? Please elaborate
5. If no to 4 above, did the government need to make any adaptations to include COVID-19 healthcare services? Please elaborate
6. Is there a defined set of COVID-19 individual healthcare services that the government is paying for on behalf of residents? What services are these?
7. What process was used to determine/develop these services?
8. Does this set of services adequately represent resident needs for COVID-19 services?
9. Have there been any challenges with availability of COVID-19 healthcare services that are paid for by the government? Please elaborate

From who to purchase

10. What types of healthcare facilities (level and ownership) does the government and government agencies (such as a social health insurer) use to provide healthcare services to its residents?
11. Was this range of healthcare facilities that the government and its agencies (such as a social health insurer) use to provide healthcare services to its residents adequate/sufficient/enough to provide COVID-19 health services? Please elaborate
12. Has the government and its agencies (such as a social insurer) made any changes on the type of healthcare facilities it uses to provide healthcare services, so that it can effectively provide COVID-19 services to its residents? Please elaborate
13. What types of health facilities (ownership and levels) is the government and its agencies using to provide COVID-19 healthcare services to its residents?
14. What contractual agreements exist between these facilities and the government and its agencies (such as a social health insurer)?

15. Are there any arrangements in place for residents to seek COVID-19 healthcare services from private healthcare facilities and for government and government agencies (e.g. social insurer) to pay for the services?
16. What is the experience of both the private sector and government with this arrangement?
17. What challenges have been faced with the range, type, and ownership of healthcare facilities that the government and its agencies are using to provide healthcare services to residents?

How to purchase

18. How does the government or its agencies (e.g. a social health insurer) usually pay for healthcare services (e.g. through line item budgets, global budgets, capitation, fee for services etc)?
19. For each of the methods you describe, what specific services is it used to pay for?
20. In your view, were these payment methods adequate/sufficient/appropriate for use to pay for COVID-19 services? Please elaborate
21. Did the government or its agencies have to introduce new payment methods to be used for COVID-19 healthcare services?
22. What payment methods does the government and its agencies (e.g. social insurer) use to pay for COVID-19 individual services (testing, isolation, treatment etc.)?
23. What specific service is paid for by a specific method? Why was this mechanism chosen?
24. What challenges have been faced with the method the government and its agencies are using to pay healthcare facilities for COVID-19 healthcare services?

1. How have these workarounds/adaptations impacted on fiscal responsibility, efficiency, and accountability of budget formulation, execution, and oversight of PFM processes during COVID response?

B. <u>Examining how COVID-19 and the country's response has affected the financing for RMNCAH services in LMIC</u>

1. How has COVID-19 and its containment measures affected the funding for RMNCAH?
2. How has COVID-19 and its containment measures affected the funding for family planning (FP)?
3. Has there been any reallocation/reprogramming of domestic funds for RMNCAH to COVID-19?
4. Has there been any reallocation of domestic funds for FP to COVID-19?
5. Has there been any reallocation/reprogramming of donor funds for RMNCAH to COVID-19?
6. Has there been any reallocation of donor funds for FP to COVID-19?
7. If financing for RMNCAH has been affected, how has this affected delivery of RMNCAH?
8. If financing for FP has been affected, how has this affected delivery of FP services?
9. What measures/adaptations have you put in place to minimize the negative impact of COVID-19 and its containment measures on MNCAH funding?
10. What measures/adaptations have you put in place to minimize the negative impact of COVID-19 and its containment measures on FP funding?

C. <u>Examining how COVID-19 has impacted on LMIC health financing system and affected the longer-term plans for UHC</u>

1. Has your country made any formal commitment to achieve UHC? Describe this commitment
2. Is your country implementing or planning to implement a UHC programme? Please describe this
3. How has COVID-19's economic impact affected the availability of public resources for UHC?
4. How has the pandemic experience affected (positively or negatively or not) the prioritization of UHC as a health sector goal?
5. How have/or could the country response to the pandemic (such as adapting various systems and processes) be beneficial to country approaches to UHC?
6. Has there been reallocation of resources from essential services to COVID-19, if yes, please give examples
7. If yes to 6, how has this affected the resources that are available for other essential health services?
8. What lessons can be drawn from the COVID-19 experience to inform the future design of health financing arrangements?
